# Automated detection of intracranial large vessel occlusions using Viz.ai software: Experience in a large, integrated stroke network

**DOI:** 10.1002/brb3.2808

**Published:** 2022-12-01

**Authors:** Rahul R. Karamchandani, Anna Maria Helms, Sagar Satyanarayana, Hongmei Yang, Jonathan D. Clemente, Gary Defilipp, Dale Strong, Jeremy B. Rhoten, Andrew W. Asimos

**Affiliations:** ^1^ Neurology, Neurosciences Institute Atrium Health Charlotte North Carolina USA; ^2^ Neurosciences Institute Atrium Health Charlotte North Carolina USA; ^3^ Information and Analytics Services Atrium Health Charlotte North Carolina USA; ^4^ Charlotte Radiology, Neurosciences Institute Atrium Health Charlotte North Carolina USA; ^5^ Emergency Medicine, Neurosciences Institute Atrium Health Charlotte North Carolina USA

**Keywords:** artificial intelligence, large vessel occlusion, Viz.ai

## Abstract

**Background and purpose:**

Endovascular thrombectomy is an evidence‐based treatment for large vessel occlusion (LVO) stroke. Commercially available artificial intelligence has been designed to detect the presence of an LVO on computed tomography angiogram (CTA). We compared Viz.ai‐LVO (San Francisco, CA, USA) to CTA interpretation by board‐certified neuroradiologists (NRs) in a large, integrated stroke network.

**Methods:**

From January 2021 to December 2021, we compared Viz.ai detection of an internal carotid artery (ICA) or middle cerebral artery first segment (MCA‐M1) occlusion to the gold standard of CTA interpretation by board‐certified NRs for all code stroke CTAs. On a monthly basis, sensitivity, specificity, accuracy, positive predictive value (PPV), and negative predictive value (NPV) were calculated. Trend analyses were conducted to evaluate for any improvement of LVO detection by the software over time.

**Results:**

3851 patients met study inclusion criteria, of whom 220 (5.7%) had an ICA or MCA‐M1 occlusion per NR. Sensitivity and specificity were 78.2% (95% CI 72%–83%) and 97% (95% CI 96%–98%), respectively. PPV was 61% (95% CI 55%–67%), NPV 99% (95% CI 98%–99%), and accuracy was 95.9% (95% CI 95.3%–96.5%). Neither specificity or sensitivity improved over time in the trend analysis.

**Conclusions:**

Viz.ai‐LVO has high specificity and moderately high sensitivity to detect an ICA or proximal MCA occlusion. The software has the potential to streamline code stroke workflows and may be particularly impactful when emergency access to NRs or vascular neurologists is limited.

## Graphical abstract

Commercially available artificial intelligence has been designed to detect the presence of a large vessel occlusion (LVO) stroke on computed to mography angiogram. In our analysis of 3851 patients from a large, integrated health system, Viz.ai‐LVO had high specificity and moderately high sensitivity to detect an internal carotid artery or proximal middle cerebral artery occlusion. The software has the potential to stream line code stroke work flows and may be particularly impactful when emergency access to neuro radiologists or vascular neurologists is limited.

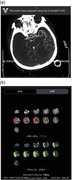

## 1 INTRODUCTION

Large vessel occlusion (LVO) stroke is associated with disproportionately higher morbidity and mortality compared to other stroke types (Malhotra et al., 2017). Endovascular thrombectomy (EVT) is an evidence‐based treatment for LVO stroke that has been demonstrated to improve functional outcomes in multiple randomized trials (Albers et al., 2018; Goyal et al., 2016; Nogueira et al., 2018). Additionally, earlier revascularization of an LVO results in better patient outcomes (Jahan et al., 2019).

The first step toward expeditious treatment is timely identification of an LVO, and various commercially available artificial intelligence (AI) software programs incorporate technology for this purpose (Murray et al., 2020; Soun et al., 2021). Prior groups have reported high sensitivity and specificity for LVO detection compared to human interpretation (Barreira et al., 2018; Murray et al., 2020), though others have yielded reduced ability to rule in or rule out an LVO with AI (Öman et al., 2019).

Given the importance of both accurate and timely identification of an LVO, we studied the performance of an increasingly utilized AI resource, Viz.ai (San Francisco, California, USA), in a large, integrated stroke network. We compared Viz.ai's automated LVO detection application to the gold‐standard computed tomography angiogram (CTA) interpretation of board‐certified neuroradiologists. Quantifying the ability of Viz.ai to detect LVOs can identify software limitations that clinicians need to be mindful of while caring for the acute stroke patient and streamline code stroke workflows.

## 2 METHODS

### 2.1 Patient population and measurements of interest

From a large, integrated health system that evaluates over 6000 code strokes annually, we conducted a retrospective analysis from a prospectively collected code stroke registry of patients presenting between January 2021 and December 2021. Consecutive code stroke CTAs were sent to Viz.ai for analysis based on imaging protocols for the health system, which require obtaining an unenhanced CT, CTA, and CT perfusion (CTP) scan for patients presenting with cortical signs in the 0–24 h window (Hoglund et al., 2020).

The study was approved by the Institutional Review Board (IRB) of the hub hospital of the health system, a Joint Commission‐Sponsored Comprehensive Stroke Center. Due to the retrospective study design, the requirement for informed consent was waived by the IRB. The data that supports the findings of this study are available from the corresponding author upon reasonable request.

The gold standard for LVO detection was CTA interpretation by a board‐certified, fellowship‐trained neuroradiologist. Interpreting neuroradiologists were provided with the presenting history by the treating clinical team and also read the corresponding unenhanced CT scan. Automated LVO detection using a convolutional neural network with Viz.ai version 1.84.0 was compared to formal neuroradiology (NR) CTA interpretation. In the primary analysis, the performance of Viz.ai‐LVO for detecting internal carotid artery (ICA) or middle cerebral artery, first segment (MCA‐M1) occlusions was assessed. The secondary analysis assessed the ability of Viz.ai‐LVO to detect ICA, MCA‐M1, or MCA proximal second segment (MCA‐M2) occlusions.

True positives (TP) were defined as Viz.ai‐LVO detection of an LVO as per the gold standard of NR CTA interpretation. Distal MCA branch occlusions, alternate vessel occlusions (i.e., anterior cerebral artery [ACA] or basilar artery), or high‐grade stenosis, were not considered LVOs. True negatives (TN) were the absence of LVO detection by Viz.ai that agreed with NR. False positives (FP) were the detection of an LVO by Viz.ai that was not present on formal NR read, while false negatives (FN) were the absence of an LVO by Viz.ai software that was present as per NR. Sensitivity was the ratio of (true positives) / (true positives + false negatives) and specificity was the ratio of (true negatives) / (true negatives + false positives). Positive predictive value (PPV) was the ratio of (true positives) / (true positives + false positives) and negative predictive value (NPV) was (true negatives) / (true negatives + false negatives). The most common reasons for FN and FP results were tabulated for detection of ICA, MCA‐M1, and MCA‐M2 occlusions.

### 2.2 Statistical analysis

Descriptive analyses of patient demographics and clinical characteristics were reported as mean +/− standard deviation (SD) for age and total number (percentage) for sex and comorbidities (hypertension, hyperlipidemia, diabetes, atrial fibrillation, smoking), which were collected from the patient's electronic medical record.

Commonly used diagnostic testing metrics (i.e., sensitivity, specificity, PPV, NPV, area under the curve [AUC], and kappa coefficient) and their 95% confidence intervals (CIs) were reported for both the primary and secondary analyses.

Sensitivity and specificity trend analyses were conducted using linear regression to determine any significant change in software performance over the 12‐month study period.

## 3 RESULTS

Over a 12‐month study period, a total of 3851 patients met study inclusion criteria, of whom 220 (5.7%) had an ICA or MCA‐M1 occlusion per NR interpretation (Table 1). An additional 138 (3.6%) had a proximal MCA‐M2 occlusion. Demographic, patient, and radiographic characteristics are summarized in Table 1.

**TABLE 1:** Patient characteristics for all subjects

Patient characteristics	*N* = 3851
Age, mean (SD)	64.35 (15.98)
Male Sex, *n* (%)	1683 (43.7)
Hypertension, *n* (%)	2471 (64.2)
Hyperlipidemia, *n* (%)	1665 (43.2)
Diabetes, *n* (%)	1194 (31)
Atrial fibrillation, *n* (%)	485 (12.6)
Smoking, *n* (%)	1057 (27.4)
ICA occlusion, *n* (%)	63 (1.6)
MCA‐M1 occlusion, *n* (%)	157 (4.1)
MCA‐M2 occlusion, *n* (%)	138 (3.6)

Abbreviations: ICA, internal carotid artery; M1, MCA first segment; M2, MCA second segment; MCA, middle cerebral artery; *N*, total number; *n*, number; SD, standard deviation.

**FIGURE 1:**
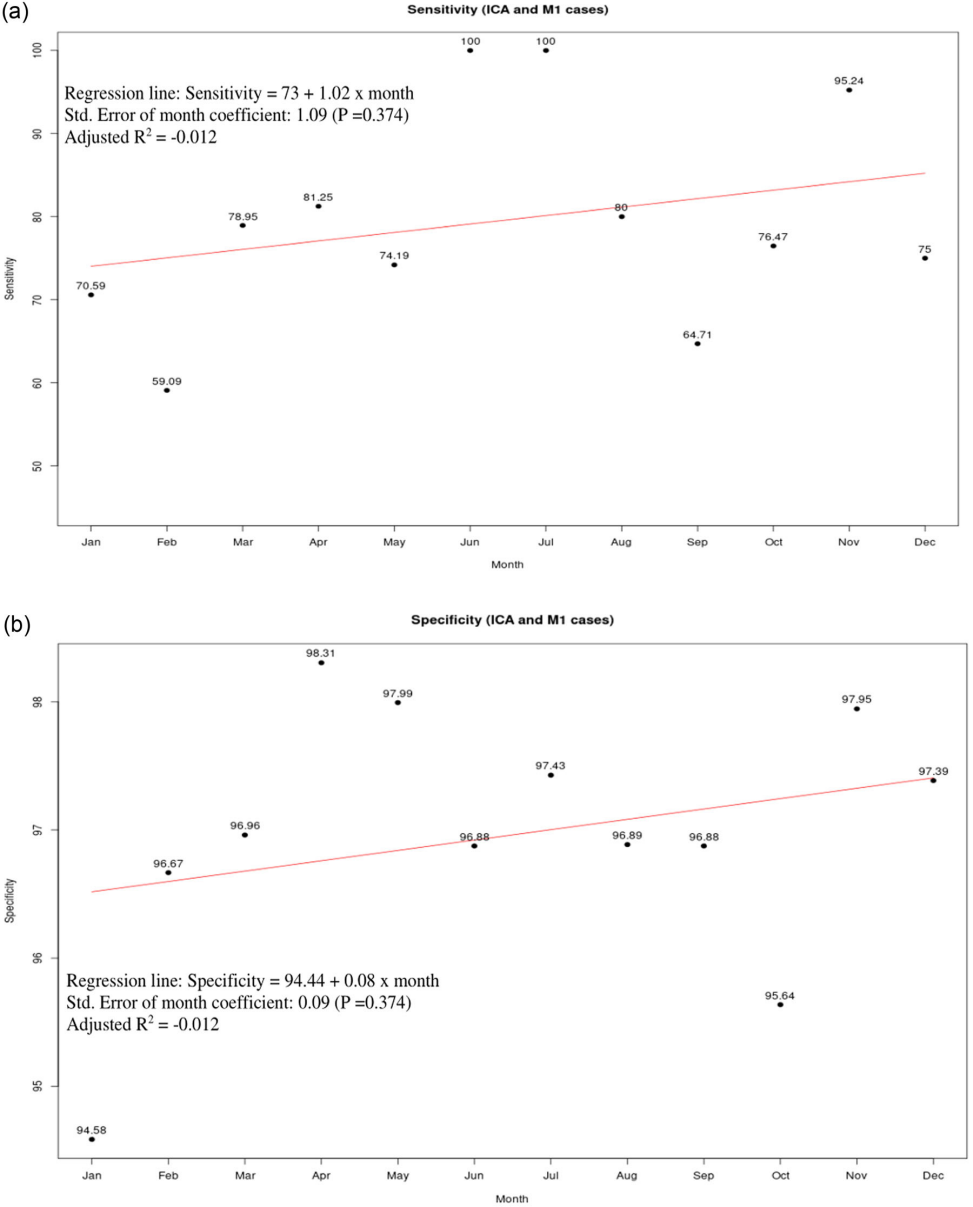
Sensitivity and specificity trend analyses for Viz.ai‐LVO detection of ICA and MCA‐M1 occlusions. No significant increase in (a) sensitivity nor (b) specificity was seen for Viz.ai‐LVO detection of ICA or MCA‐M1 occlusions during the 12‐month study period. ICA, internal carotid artery; LVO, large vessel occlusion; MCA‐M1, middle cerebral artery, first segment.

Table 2 displays the monthly performance of Viz.ai for detecting an ICA or MCA‐M1 occlusion. Sensitivity and specificity for the full 12‐month study period were 78.2% (95% CI 72%−83%) and 97% (95% CI 96%−98%), respectively (Table 2). PPV was 61% (95% CI 55%−67%), NPV 99% (95% CI 98%−99%), accuracy was 95.9% (95% CI 95.3%−96.5%), AUC 0.88 (95% CI 0.85‐0.90), and kappa's coefficient for agreement with NR interpretation was 0.67. In the trend analysis, neither sensitivity (*p* = 0.3745) nor specificity (*p* = 0.3744) demonstrated any significant improvement over the study period at the 95% CI (Figure 1).

**TABLE 2:** Primary analysis

Month	TN (*n*)	TP (*n*)	FN (*n*)	FP (*n*)	Sensitivity (%)	Specificity (%)
January	262	12	5	15	70.6	94.6
February	290	13	9	10	59.1	96.7
March	319	15	4	10	78.9	97
April	290	13	3	5	81.3	98.3
May	342	23	8	7	74.2	98
June	310	12	0	10	100	96.9
July	303	12	0	8	100	97.4
August	280	16	4	9	80	96.9
September	279	11	6	9	64.7	96.9
October	263	13	4	12	76.5	95.6
November	286	20	1	6	95.2	97.9
December	298	12	4	8	75	97.4
**Total**	3522	172	48	109	78.2	97

Performance of Viz.ai Large Vessel Occlusion Software for Detection of ICA or MCA‐M1 Occlusion.

Abbreviations: FN, false negative; FP, false positive; ICA, internal carotid artery; MCA‐M1, middle cerebral artery first segment; *n*, number; TN, true negative; TP, true positive.

**FIGURE 2:**
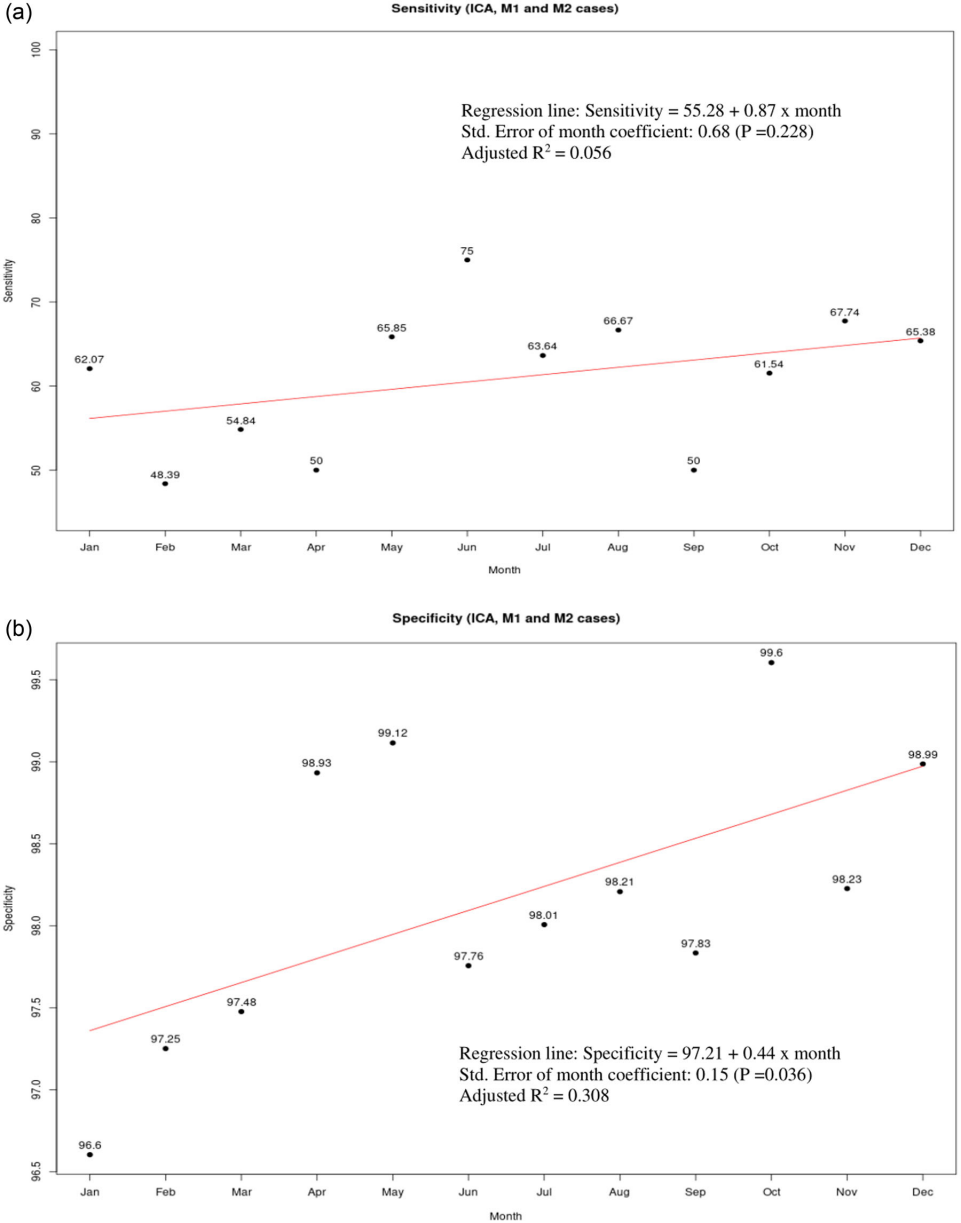
Sensitivity and specificity trend analyses for Viz.ai‐LVO detection of ICA, MCA‐M1, or proximal MCA‐M2 occlusions. No significant increase in (a) sensitivity was seen for Viz.ai‐LVO detection of ICA, MCA‐M1, or proximal MCA‐M2 occlusions during the 12‐month study period, though a statistically significant increase in (b) specificity was seen. ICA, internal carotid artery; LVO, large vessel occlusion; MCA‐M1, middle cerebral artery, first segment; MCA‐M2, middle cerebral artery, second segment.

The secondary analysis, evaluating Viz.ai‐LVO for ICA, MCA‐M1, or proximal MCA‐M2 branch occlusions, is summarized in Table 3. Notably, sensitivity was 60.6% (95% CI 55%−66%), specificity 98.2% (95% CI 98%−99%), and accuracy was 94.7% (95% CI 93.9%−95.4%). PPV was 77% (95% CI 72%−82%), NPV 96% (95% CI 95%−97%), AUC 0.79 (95% CI 0.77‐0.82), and kappa's coefficient was 0.65. Specificity significantly improved over time (*p* = 0.0357) whereas sensitivity (*p* = 0.2281) demonstrated no significant improvement at the 95% CI (Figure 2).

**TABLE 3:** Secondary analysis

Month	TN (*n*)	TP (*n*)	FN (*n*)	FP (*n*)	Sensitivity (%)	Specificity (%)
January	256	18	11	9	62.1	96.6
February	283	15	16	8	48.4	97.3
March	309	17	14	8	54.8	97.5
April	278	15	15	3	50	98.9
May	336	27	14	3	65.9	99.1
June	305	15	5	7	75	97.8
July	295	14	8	6	63.6	98
August	274	20	10	5	66.7	98.2
September	271	14	14	6	50	97.8
October	252	24	15	1	61.5	99.6
November	277	21	10	5	67.7	98.2
December	293	17	9	3	65.4	99
**Total**	3429	217	141	64	60.6	98.2

Performance of Viz.ai Large Vessel Occlusion Software for Detection of ICA, MCA‐M1, or Proximal MCA‐M2 Occlusion.

Abbreviations: FN, false negative; FP, false positive; ICA, internal carotid artery; MCA‐M1, middle cerebral artery first segment; *n*, number; TN, true negative; TP, true positive.

**FIGURE 3:**
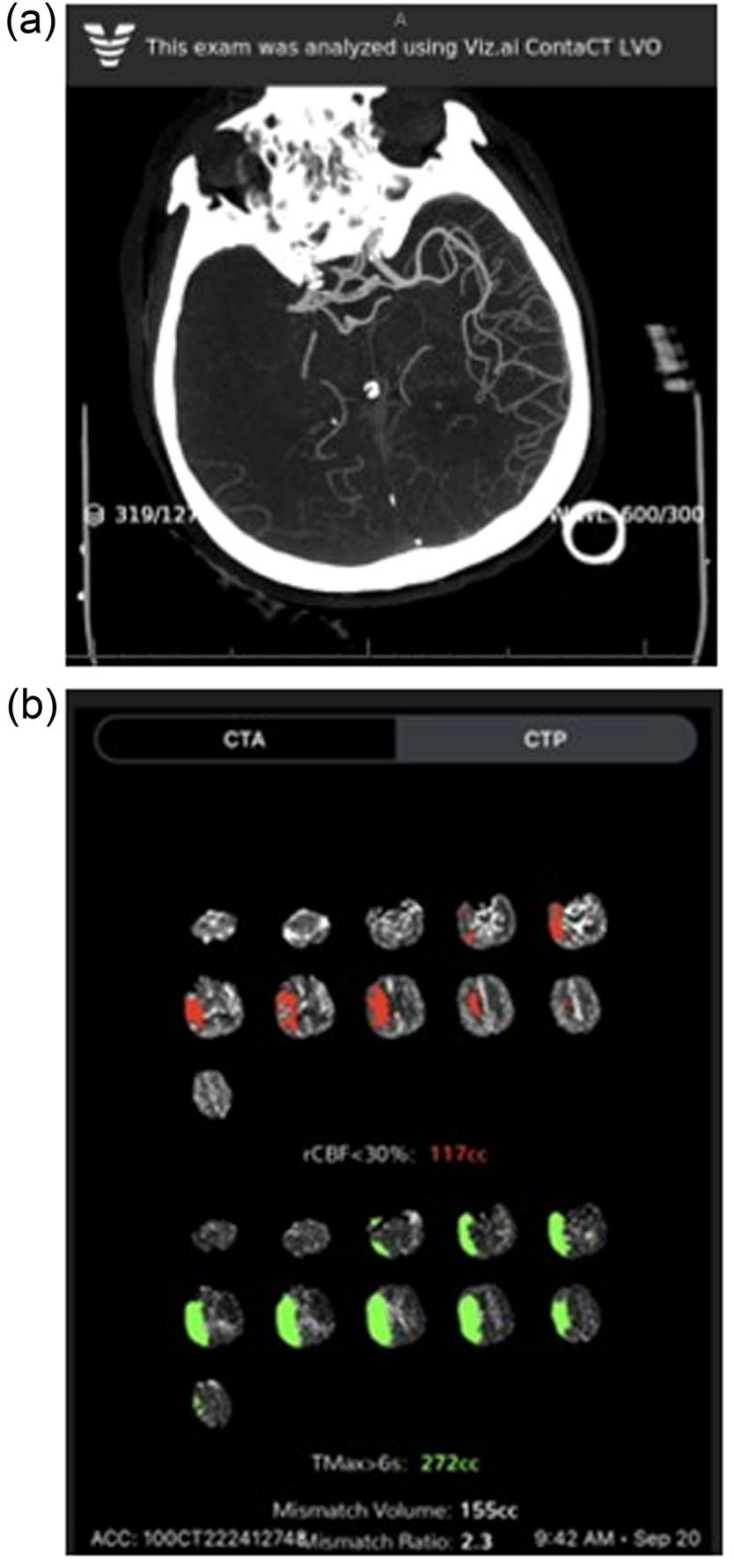
Example of Viz.ai's automated large vessel occlusion detection software and computed tomography perfusion output. (a) Automated output of Viz.ai's large vessel occlusion detection software demonstrating a proximal right middle cerebral artery (MCA)‐M1 occlusion. (b) Automated computed tomography perfusion output for the same patient showing core infarction of 117 cubic centimeters (cc) and penumbra of 155 cc in the right MCA territory.

The most common reasons for FN results in detecting ICA, MCA‐M1, or proximal MCA‐M2 occlusions included (1) a “non‐main” vessel occlusion (i.e., the algorithm failed to detect an occluded branch of the MCA); (2) incorrect vessel segmentation by the software; and (3) vascular anatomy (i.e., short segment vessel occlusion, occlusion at “end point” of branch, or occlusion “hidden” by an overlying branch). The most common reasons for FP results included (1) low Hounsfield Units (low quality scan); (2) incorrect vessel segmentation; and (3) vascular stenosis, rather than occlusion. An example of Viz.ai's automated LVO detection software and CTP output is shown in Figure 3.

## 4 DISCUSSION

We have evaluated the ability of the AI software Viz.ai to detect an ICA or proximal MCA occlusion compared to CTA interpretation by board‐certified neuroradiologists. Our analysis demonstrates consistently high specificity for LVO detection, though only moderately high sensitivity, in the largest series to date evaluating the ability of an AI platform to auto‐detect the presence of an LVO.

Automated LVO detection has the potential to streamline code stroke workflows by alerting members of the treatment team in a timelier fashion. This is especially important given that earlier revascularization of an LVO improves patient outcomes (Jahan et al., 2019). Thus, any and every effort should be made to expedite treatment. Earlier identification of an LVO allows for quicker treatment at a thrombectomy center or for more expeditious transfer to a capable center, when appropriate.

The major strength of our study is the use of registry data from a large, integrated health system. This represents real‐world practice in a variety of clinical environments, including urban and rural settings, with differing access to emergency neurology (in‐person vs. virtual), and with AI software installed on a multitude of CT scanners (Table S1). These diverse settings are a distinguishing feature of our analysis and contrast with previously published single center studies (Rodrigues et al., 2022; Vitellas et al., 2022; Yahav‐Dovrat et al., 2021).

An additional strength of our analysis is the monthly performance tracking of Viz.ai‐LVO. For the detection of ICA or MCA‐M1 occlusions, we report no significant improvement in sensitivity or specificity trend analyses. The secondary analysis, which also included detection of proximal MCA‐M2 occlusions, showed significantly improved specificity over time. We hypothesize that this may be related to periodic improvement of the machine learning algorithm.

Radiologists across all levels of training, including neuroradiologists, non‐neuroradiologists, and radiology residents, have been shown to have high sensitivity and specificity for detection of an intracranial LVO on CTA, though neuroradiologists may have faster interpretation times than their non‐NR colleagues (Boyd et al., 2020). When LVO detection was compared between neuroradiologists and a combination of vascular neurologists and general neurologists, agreement was “strong” or “very strong” with respect to detecting MCA‐M1, ICA, ACA first (A1) or second (A2) segment, or basilar artery, occlusions (Bar et al., 2017). Agreement was “moderate” for MCA‐M2 occlusions and “poor” for ICA‐terminus (ICA‐T) occlusions. As such, we opted to compare Viz.ai‐LVO detection to interpretation by board‐certified neuroradiologists, as done previously (Yahav‐Dovrat et al., 2021), and in contrast to comparisons to vascular neurologists (Barreira et al., 2018; Rodrigues et al., 2022), recognizing that hyperacute NR interpretation may not be available in all settings.

Prior groups have studied the performance of Viz.ai for LVO detection. Rodrigues et al. (2022), in a study of 610 CTAs, demonstrated high sensitivity (87.6%), specificity (88.5%), and accuracy (87.9%) compared to vascular neurologists to detect an ICA‐T or MCA‐M1 occlusion. The secondary analysis, which included detection of proximal MCA‐M2 occlusions, yielded sensitivity, specificity, and accuracy of 80.3%, 88.5%, and 87.9%, respectively. In the 875 CTAs included in another analysis, Viz.ai had a sensitivity of 90.1%, specificity of 82.5%, and accuracy of 86% compared to vascular neurologists for ICA‐T or MCA‐M1 occlusions (Barreira et al., 2018). An analysis from an academic comprehensive stroke center that included 282 patients yielded sensitivity of 90.6% and specificity of 87.6% for detection of an intracranial ICA, MCA‐M1, or MCA‐M2 occlusion (Vitellas et al., 2022). Most recently, Yahav‐Dovrat et al. (2021) reported a sensitivity of 81%, specificity of 96%, and accuracy of 94% compared to neuroradiologists to detect ICA or MCA‐M1 occlusions.

The notable distinction in our findings compared to previous studies is reduced sensitivity for Viz.ai LVO detection. There are several potential explanations for this. First, our analysis includes a large sample size and represents a real‐world experience of CTP acquisition in an integrated hub and spoke network of hospitals, rather than at a single center (Rodrigues et al., 2022; Vitellas et al., 2022; Yahav‐Dovrat et al., 2021) or multiple tertiary centers (Barreira et al., 2018). Thus, technical differences between CT scanners or differences in CTP acquisition protocols may have influenced our findings. Additionally, as all CTAs in our analysis were compared to interpretation by board‐certified, fellowship‐trained neuroradiologists, rather than vascular neurologists (Barreira et al., 2018; Rodrigues et al., 2022) or general diagnostic radiologists, this consequently could have influenced our results compared to other groups. Moreover, alternate versions of Viz.ai's software have the potential to impact LVO detection capabilities, and these versions were not always specified in prior reports (Vitellas et al., 2022). Despite these differences in study methodology, we view the consistently high specificity for LVO detection with Viz.ai as an opportunity to alert stroke teams on a more urgent basis. This can be leveraged most in health systems that do not have access to hyperacute, formal NR interpretation, or vascular‐trained neurologists. However, the only moderate sensitivity for LVO detection in our study does not allow the clinician to confidently “rule out” an ICA or proximal MCA occlusion, so the absence of an alert from the AI, particularly in cases of clinical stroke syndromes or CTP patterns that suggest the presence of an LVO, should still be managed with vigilance and careful examination of the CTA.

A recent study evaluated the ability of an alternate AI source, StrokeViewer (Amsterdam, The Netherlands), to detect ICA or MCA‐M1 occlusions in a sample of 474 patients (Fasen et al., 2022). Sensitivity was 77.3%, specificity 88.5%, and accuracy was 95.2%. Accuracy was lower for MCA‐M2 detection (18 of 33, 54.5%). Sensitivity with StrokeViewer was comparable to our findings, though specificity was notably lower, and accuracy was similar. Unsurprisingly, accuracy was lower for MCA‐M2 occlusions (Fasen et al., 2022), similar to our results and a previous report (Rodrigues et al., 2022). These findings highlight the need for AI refinement to more accurately identify distal branch occlusions, particularly given the mounting data that suggest that thrombectomy for patients with MCA‐M2 occlusions is safe and effective (Cho & Choi, 2021). Fasen et al. demonstrate that alternate AI technologies also have the potential to streamline code stroke workflows, if the limitations of each software program are understood. Future efforts may focus on not only detection of more distal branch occlusions, but also short segment occlusions, and AI improvements in vascular segmentation, as identified in our analysis as the most common reasons for FN results. Additional AI optimization may also address differentiation of high‐grade vascular stenoses from occlusions, a common cause of FP alerts in our series.

Besides LVO detection, AI software has been developed to provide additional diagnostic information for the acute stroke patient (Soun et al., 2021). This includes automation of early ischemic change in the MCA territory with the Alberta Stroke Program Early CT Score, quantification of infarct core size and penumbra, collateral circulation scoring, hemorrhage detection and quantification, aneurysm measurements, and triage and notification systems (Soun et al., 2021). In addition to Viz.ai and StrokeViewer, popular commercially available AI platforms for acute stroke imaging include RAPID AI (iSchemaView, Menlo Park, CA, USA), Aidoc (Tel Aviv, Israel), Brainomix (Oxford, United Kingdom), and Avicenna.AI (La Ciotat, Provence‐Alpes‐Cote d'Azur, France) (Soun et al., 2021).

Our study has several limitations. The data were collected monthly, though retrospectively, so they are subject to the inherent limitations of a retrospective analysis. NR interpretation was used from the written report, and thus may have differed from the stat “wet read” provided to the clinical team (i.e., a distal branch occlusion may have been later noted that was not initially seen on the stat read provided to the clinical team). A single neuroradiologist read each CTA, so inter‐rater reliability between NR reads could not be calculated. Neuroradiologists were provided with clinical information from the patient's presenting history, and interpreted a patient's corresponding unenhanced CT scan, so these factors may have served as a source of bias. FP scans, although not consistent with an ICA or proximal MCA occlusion, may have still provided valuable information in the acute setting (i.e., distal MCA branch occlusion, cervical or intracranial stenosis). The PPV and NPV rates are dependent on the prevalence of an LVO in the CTAs sent to Viz.ai for analysis, which may differ among health systems based on screening protocols.

In summary, in the largest series reported to date, we have evaluated Viz.ai's LVO detection software versus formal neuroradiology CTA interpretation. We report a consistently high specificity, and thus high confidence for ruling in an ICA or MCA‐M1 occlusion. Sensitivity was only moderately high, and lower than previously reported, which may be the result of our large sample size; technical differences between CT scanners, technicians, or CTP acquisition protocols; different versions of Viz.ai; or comparison to board‐certified NR interpretation. Earlier stroke team notification of an LVO with AI offers an opportunity for expedited treatment and may be particularly valuable to emergency clinicians in the absence of access to neuroradiologists or vascular neurologists. Future efforts may focus on the ability of AI to detect more distal branch occlusions, short segment occlusions, posterior circulation occlusions, and other clinically relevant vascular findings for the acute stroke patient.

## CONFLICT OF INTEREST

The authors declare no conflict of interest.

### PEER REVIEW

The peer review history for this article is available at https://publons.com/publon/10.1002/brb3.2808

## Supporting information

Supplemental Table 1. CT Scanners and Acquisition Protocols

## Data Availability

The data that supports the findings of this study are available from the corresponding author upon reasonable request.

## References

[brb32808-bib-0001] Albers, G. W. , Marks, M. P. , Kemp, S. , Christensen, S. , Tsai, J. P. , Ortega‐Gutierrez, S. , McTaggart, R. A. , Torbey, M. T. , Kim‐Tenser, M. , Leslie‐Mazwi, T. , Sarraj, A. , Kasner, S. E. , Ansari, S. A. , Yeatts, S. D. , Hamilton, S. , Mlynash, M. , Heit, J. J. , Zaharchuk, G. , Kim, S. , … Lansberg, M. G. (2018). Thrombectomy for stroke at 6 to 16 hours with selection by perfusion imaging. New England Journal of Medicine, 378, 708–718. 29364767 10.1056/NEJMoa1713973 PMC6590673

[brb32808-bib-0002] Bar, M. , Kral, J. , Jonszta, T. , Marcian, V. , Kuliha, M. , & Mikulik, R. (2017). Interrater variability for CT angiography evaluation between neurologists and neuroradiologist in acute stroke patients. British Journal of Radiology, 90, 20160670. 28118025 10.1259/bjr.20160670 PMC5601522

[brb32808-bib-0003] Barreira, C. , Bouslama, M. , Lim, J. , Al‐Bayati, A. , Saleem, Y. , Devlin, T. , Haussen, D. , Froehler, M. , Grossberg, J. , Baxter, B. , Frankel, M. , & Nogueira, R. (2018). E‐108 Aladin study: Automated large artery occlusion detection in stroke imaging study – A multicenter analysis. Journal of NeuroInterventional Surgery, 10, A101–A102.

[brb32808-bib-0004] Boyd, C. A. , Jayaraman, M. V. , Baird, G. L. , Einhorn, W. S. , Stib, M. T. , Atalay, M. K. , Boxerman, J. L. , Lourenco, A. P. , Jindal, G. , Hidlay, D. T. , DiBiasio, E. L. , & McTaggart, R. A. (2020). Detection of emergent large vessel occlusion stroke with CT angiography is high across all levels of radiology training and grayscale viewing methods. European Radiology, 30, 4447–4453. 32232790 10.1007/s00330-020-06814-9

[brb32808-bib-0005] Cho, Y.‐H. , & Choi, J. H. (2021). Mechanical thrombectomy for acute ischemic stroke with occlusion of the M2 segment of the middle cerebral artery: A literature review. Journal of Cerebrovascular and Endovascular Neurosurgery, 23, 193–200. 34492752 10.7461/jcen.2021.E2020.11.002 PMC8497726

[brb32808-bib-0006] Fasen, B. , Berendsen, R. C. M. , & Kwee, R. M. (2022). Artificial intelligence software for diagnosing intracranial arterial occlusion in patients with acute ischemic stroke. Neuroradiology, 64, 1579–1583. 35137270 10.1007/s00234-022-02912-1

[brb32808-bib-0007] Goyal, M. , Menon, B. K. , van Zwam, W. H. , Dippel, D. W. , Mitchell, P. J. , Demchuk, A. M. , Dávalos, A. , Majoie, C. B. , van der Lugt, A. , & De Miquel, M. A. , Donnan, G. A. , Roos, Y. B. W. E. M. , Bonafe, A. , Jahan, R. , Diener, H.‐C. , van de Berg, L. A. , Levy, E. I. , Berkhemer, O. A. , Pereria, V. M. , … Jovin, T. G. , HERMES collaborators . (2016). Endovascular thrombectomy after large‐vessel ischaemic stroke: A meta‐analysis of individual patient data from five randomised trials. Lancet, 387, 1723–1731. 26898852 10.1016/S0140-6736(16)00163-X

[brb32808-bib-0008] Hoglund, J. , Strong, D. , Rhoten, J. , Chang, B. , Karamchandani, R. , Dunn, C. , Yang, H. , & Asimos, A. W. (2020). Test characteristics of a 5‐element cortical screen for identifying anterior circulation large vessel occlusion ischemic strokes. Journal of the American College of Emergency Physicians Open, 1, 908–917. 33145539 10.1002/emp2.12188 PMC7593424

[brb32808-bib-0009] Jahan, R. , Saver, J. L. , Schwamm, L. H. , Fonarow, G. C. , Liang, L. , Matsouaka, R. A. , Xian, Y. , Holmes, D. N. , Peterson, E. D. , Yavagal, D. , & Smith, E. E. (2019). Association between time to treatment with endovascular reperfusion therapy and outcomes in patients with acute ischemic stroke treated in clinical practice. JAMA, 322, 252–263. 31310296 10.1001/jama.2019.8286 PMC6635908

[brb32808-bib-0010] Malhotra, K. , Gornbein, J. , & Saver, J. L. (2017). Ischemic strokes due to large‐vessel occlusions contribute disproportionately to stroke‐related dependence and death: A review. Frontiers in Neurology, 8, 651. 29250029 10.3389/fneur.2017.00651 PMC5715197

[brb32808-bib-0011] Murray, N. M. , Unberath, M. , Hager, G. D. , & Hui, F. K. (2020). Artificial intelligence to diagnose ischemic stroke and identify large vessel occlusions: A systematic review. Journal of NeuroInterventional Surgery, 12, 156–164. 31594798 10.1136/neurintsurg-2019-015135

[brb32808-bib-0012] Nogueira, R. G. , Jadhav, A. P. , Haussen, D. C. , Bonafe, A. , Budzik, R. F. , Bhuva, P. , Yavagal, D. R. , Ribo, M. , Cognard, C. , Hanel, R. A. , Sila, C. A. , Hassan, A. E. , Millan, M. , Levy, E. I. , Mitchell, P. , Chen, M. , English, J. D. , Shah, Q. A. , Silver, F. L. , …, & Jovin, T. G. (2018). Thrombectomy 6 to 24 hours after stroke with a mismatch between deficit and infarct. New England Journal of Medicine, 378, 11–21. 29129157 10.1056/NEJMoa1706442

[brb32808-bib-0013] Öman, O. , Mäkelä, T. , Salli, E. , Savolainen, S. , & Kangasniemi, M. (2019). 3D convolutional neural networks applied to CT angiography in the detection of acute ischemic stroke. European Radiology Experimental, 3, 8. 30758694 10.1186/s41747-019-0085-6 PMC6374492

[brb32808-bib-0014] Rodrigues, G. , Barreira, C. M. , Bouslama, M. , Haussen, D. C. , Al‐Bayati, A. , Pisani, L. , Liberato, B. , Bhatt, N. , Frankel, M. R. , & Nogueira, R. G. (2022). Automated large artery occlusion detection in stroke: A single‐center validation study of an artificial intelligence algorithm. Cerebrovascular Diseases, 51, 259–264. 34710872 10.1159/000519125

[brb32808-bib-0015] Soun, J. E. , Chow, D. S. , Nagamine, M. , Takhtawala, R. S. , Filippi, C. G. , Yu, W. , & Chang, P. D. (2021). Artificial intelligence and acute stroke imaging. American Journal of Neuroradiology, 42, 2–11. 33243898 10.3174/ajnr.A6883 PMC7814792

[brb32808-bib-0016] Vitellas, C. A. , Mannix, N. C. , Nimjee, S. M. , Shujaat, M. T. , Heaton, S. , & Lee, V. H. (2022). Abstract 130: Real world experience with Viz.AI automated large vessel occlusion detection. Stroke; A Journal of Cerebral Circulation, 53, A130–A130.

[brb32808-bib-0017] Yahav‐Dovrat, A. , Saban, M. , Merhav, G. , Lankri, I. , Abergel, E. , Eran, A. , Tanne, D. , Nogueira, R. G. , & Sivan‐Hoffmann, R. (2021). Evaluation of artificial intelligence–powered identification of large‐vessel occlusions in a comprehensive stroke center. American Journal of Neuroradiology, 42, 247–254. 33384294 10.3174/ajnr.A6923 PMC7872164

