# Supplementary material for: Automated detection of intracranial large vessel occlusions using Viz.ai software: Experience in a large, integrated stroke network
Source: Brain Behav. 2022 Dec 1;13(1):e2808. doi: 10.1002/brb3.2808 (PMC9847593; doi:10.1002/brb3.2808)
Supplement: Supplementary file 1 — Supplemental Table 1. CT Scanners and Acquisition Protocols [file BRB3-13-e2808-s001.docx]

Supplemental Table 1. CT Scanners and Acquisition Protocols

| Brand | Scanner | Acq. | Scan Type | Rotation Time (s) | Configuration | Coverage (mm) | ST (mm) | Interval (mm) | kV | mA | Phantom (cm) |
| --- | --- | --- | --- | --- | --- | --- | --- | --- | --- | --- | --- |
| GE | CT750 HD | 1 | Axial - S | 0.4 | 64 x 0.625 (8i) | 40 | 5.0 | 40.0 | 80 | 500 | 16 |
| GE | EVO 32 | 1 | Axial - S | 0.5 | 32 x 1.25 (8i) | 40 | 5.0 | 40.0 | 80 | 400 | 16 |
| GE | EVO 64 | 1 | Axial - S | 0.5 | 64 x 0.625 (8i) | 40 | 5.0 | 40.0 | 80 | 400 | 16 |
| GE | Optima CT660 | 1 | Axial - S | 0.5 | 32 x 1.25 (8i) | 40 | 5.0 | 40.0 | 80 | 400 | 16 |
| GE | Revolution Apex | 1 |  |  |  |  |  |  |  |  |  |
| GE | VCT | 1 | Axial - S | 0.4 | 64 x 0.625 (8i) | 40 | 5.0 | 40.0 | 80 | 500 | 16 |

Acq., acquisition; s, seconds; mm, millimeters; ST, slice thickness; kV, kilovolts; mA, milliamps; cm, centimeters; GE, General Electric. CT scanner names are as listed in the Table.
